# Evaluation of Maternal Factors Affecting Postpartum Insulin Resistance Markers in Mothers with Gestational Diabetes—A Case–Control Study

**DOI:** 10.3390/nu16223871

**Published:** 2024-11-13

**Authors:** Karolina Karcz, Paulina Gaweł, Barbara Królak-Olejnik

**Affiliations:** Department of Neonatology, Wroclaw Medical University, 50-367 Wroclaw, Poland; pszczygiol@usk.wroc.pl (P.G.); barbara.krolak-olejnik@umw.edu.pl (B.K.-O.)

**Keywords:** gestational diabetes, insulin resistance, insulin resistance markers, hypothyroidism, gestational weight gain

## Abstract

Background: Gestational diabetes mellitus (GDM) is defined by an insufficient insulin response to counteract the insulin resistance (IR) that arises from the physiological adaptations associated with pregnancy. However, the pathophysiology of IR is complex and unclear, as it encompasses elements such as epigenetics, environmental factors, modifiable lifestyle factors, and psychosocial factors. Aim: The objective of this study was to evaluate the influence of GDM and other maternal factors on IR markers in comparison to mothers with normal glucose tolerance during pregnancy in the first week postpartum. Material and Methods: The study population comprised 70 participants, including mothers with gestational diabetes who were treated with a diet and physical activity (GDM G1), with insulin (GDM G2), and a control group of healthy mothers without gestational diabetes (non-GDM). A series of statistical techniques were employed to facilitate the comparison of data between the study groups, with the objective of identifying potential associations with maternal factors. A taxonomic analysis was conducted using the following factors: classification by study group, a history of hypothyroidism in the maternal medical interview, and maternal gestational weight gain, which were identified as the best-fitting predictors. Results: The analysis resulted in the identification of four clusters of patients. Comparison of the insulin resistance markers between mothers assigned to the abovementioned clusters showed differences in the incidence of excessive weight loss and in the results of glucose screening tests during pregnancy. Also, differences concerning fasting glucose levels in the first and second/third trimesters of pregnancy and glucose levels at 1 h post-OGTT were found. For the clusters, the results of the HOMA-IR and the QUICKI did not show any differences in the first week after delivery (*p* > 0.05). HbA1c results varied significantly. Conclusions: Degree of glucose metabolism disorders, hypothyroidism, and weight gain in pregnancy influence maternal insulin resistance markers in the first week postpartum. Additionally, gestational weight fluctuation has a significant influence on the outcome of pregnancy, particularly with regard to fetal growth and, consequently, the infant’s birth weight and adipose tissue accumulation.

## 1. Introduction

Gestational diabetes mellitus (GDM) is defined as “diabetes first diagnosed in the second or third trimester of pregnancy that is not clearly overt diabetes prior to gestation” and is currently the most prevalent pregnancy complication, affecting 5.4% of European women and 3.4% of Polish women. [1,2]. The prevalence of GDM is constantly increasing with the escalating obesity epidemic, which is fueled by poor dietary habits and sedentary lifestyles [3]. 

Insulin sensitivity changes during pregnancy in order to accommodate the growth of the fetus and to meet its metabolic requirements for energy. During the physiological course of pregnancy, due to circulating placental hormones and cytokines that promote insulin resistance, maternal tissue becomes increasingly insensitive to insulin. These changes are overcome by increased pancreatic β-cell insulin secretion. If pregnant women fail to adapt to these changes, GDM as a result of inadequate insulin response to compensate for the insulin is present. However, the etiology of GDM is complex and multifactorial (epigenetics, genetics, environmental, and modifiable lifestyle factor) and remains unclear [4].

GDM is linked to both immediate and long-term adverse outcomes for both the mother and child. In the short term, women with GDM are more likely to develop hypertension and preeclampsia during pregnancy [5,6]. While in the long term, there is an increased risk of developing type-2 diabetes mellitus and cardiovascular disease (CVD) [7,8]. The newborns of diabetic mothers are observed to be born in a large-for-gestational age (LGA) and macrosomic. Additionally, the likelihood of complications during delivery is heightened, encompassing neonatal hypoglycemia, polyhydramnios, perinatal mortality, intrapartum injuries, and operative deliveries [6,9]. Furthermore, maternal diabetes during pregnancy is associated with an increased risk of developing overweight or obesity, impaired glucose tolerance, diabetes, or metabolic disease in the future. [10,11]. Among the risk factors for the development of insulin resistance and GDM, high pre-pregnancy body mass index (pBMI) and excessive weight gain during pregnancy are listed. However, GDM may also occur when normal pre-pregnancy BMI and appropriate gestational weight gain are observed. Because of the complications caused by GDM and health concerns about both mothers and her offspring, the diagnosis of GDM should be confirmed as early as possible [12]. Also, the knowledge of factors that have a major and strong impact on the course of gestational diabetes should be persistently investigated in order to improve pregnancy outcomes.

Following childbirth, insulin resistance typically declines rapidly in most GDM women, though the rate of this decline can vary depending on individual factors. In light of the extant literature, the mean time for the normalization of insulin and glucose levels, and thus the reduction in insulin resistance, is approximately a few weeks to two to three months following delivery [13,14,15]. In women who have not been diagnosed with gestational diabetes or other metabolic disorders, insulin resistance can be restored to its pre-pregnancy state within a few weeks [13,15]. The risk of insulin resistance persisting after childbirth is higher among women who are overweight or obese, as well as those who experienced insulin resistance prior to pregnancy. In such cases, complete resolution of insulin resistance may not occur, and the condition may persist for a longer period of time [14,16]. Although surrogate IR markers are not a standard test used directly to predict perinatal complications, insulin resistance indirectly affects the risk of perinatal complications [17,18]. The existing research indicates that insulin resistance in the second trimester is associated with an increased susceptibility to pregnancy-induced hypertension and fetal macrosomy [16,19]. However, it has not been established whether a similar relationship exists between insulin resistance levels in the first week following childbirth and the aforementioned perinatal complications. In addition, the precise impact of multiple maternal factors on insulin resistance levels and the frequency of its recurrence postpartum, as well as its correlation with perinatal outcomes, remain unclear.

Accordingly, the present study sought to elucidate the impact of maternal factors on insulin resistance markers in the first week after childbirth in women who developed gestational diabetes mellitus in comparison to those with normal glucose tolerance during pregnancy.

## 2. Materials and Methods

### 2.1. Study Design

**Figure 1:**
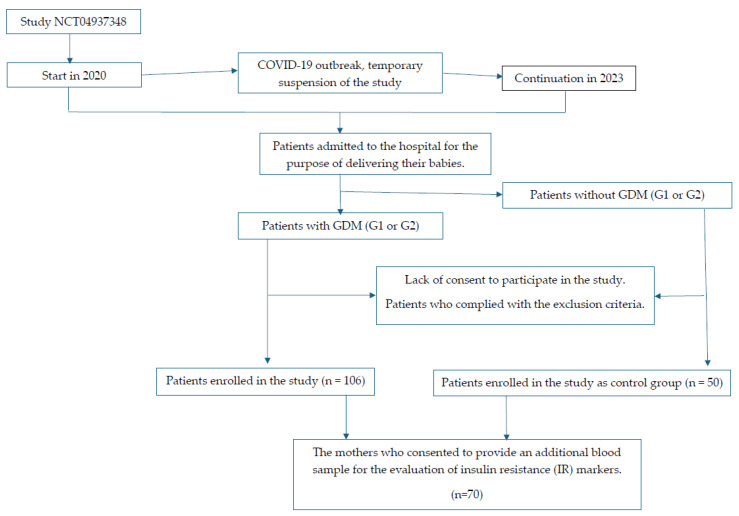
Flowchart of the patient recruitment process.

Our prospective observational case–control study was first initiated in 2020, but due to the COVID-19 pandemic and low participants, enrollment was suspended. Now, after restarting this study within 2023, the present pilot study shows initial results of insulin resistance markers in 70 of the enrolled mothers [20,21]. It should be noted that the determination of markers of insulin resistance in maternal blood was considered an optional component of this study. The objective was to ascertain the exponents of insulin resistance at the earliest possible stage following birth, namely immediately upon commencement of this study. Patients were at liberty to refuse to have their blood drawn while nevertheless consenting to the other points and assumptions of the project. The flowchart illustrating the process of patient enrollment is presented in Figure 1. All the women were invited to participate in a follow-up study, the results of which are not currently included in the manuscript.

This study was granted ethical approval by the Bioethics Committee of the Medical University of Wroclaw (KB 950/2022, dated 21 December 2022, in reference to the preceding protocols: KB 773/2019, dated 25 November 2019, KB 35/2020, dated 16 January 2020, 407/2020, dated 23 June 2020). This study was conducted according to the Declaration of Helsinki. All mothers gave written informed consent before study procedures were performed. The presented research results were conducted within the framework of the Clinical Trials Registry (https://clinicaltrials.gov/ (accessed on 18 May 2021)), NCT04937348.

### 2.2. Study Group

A total of 70 women were involved in the current study, of whom 50 were mothers with GDM. Moreover, 21 mothers in GDM were treated with diet and exercise, whereas 29 women were administered insulin therapy. No oral treatment was administered to the mothers (e.g., metformin). The remaining 20 were enrolled as a control group with no history of glucose intolerance before or during pregnancy. The cohort of mothers was homogenous with regard to nationality, comprising exclusively Polish women, and lacked any significant ethnic diversity. The mothers were patients at the 2nd Department of Gynecology and Obstetrics, and their babies were hospitalized after birth in the Department of Neonatology at the Wroclaw Medical University [20,21]. Inclusion criteria were as follows: maternal age 18–45 years, delivery at ≥35 + 0/7 weeks of gestation, vaginal delivery or cesarean section, singleton pregnancy, good postnatal condition of the newborn measured by Apgar score > 7 points, exclusive or predominant breastfeeding, mothers’ informed consent, recruitment within the first 7 days after birth. Exclusion criteria: any maternal (e.g., undernutrition, thyrotoxicosis, adrenal disorders, renal insufficiency) or neonatal condition (e.g., respiratory distress, congenital defects of cardiovascular or gastrointestinal system, necessity to start parenteral feeding, perinatal asphyxia, genetic disorders—trisomy of 21, 18, 13 chromosome, Prader–Willy syndrome, Silver–Russel syndrome, etc.) which would adversely affect the nutritional status of the newborn, maternal or neonatal congenital metabolic disease (e.g., urea cycle defects, organic or amino acids disorders), maternal abuse of alcohol, nicotine and other psychoactive substances, maternal asthma (uncontrolled, with often exacerbation, or requiring oral corticosteroid use), lack of perinatal medical care. Women were going to be excluded from the study if they had pre-existing diabetes (type 1 or 2 or other forms—e.g., LADA and MODY) or were receiving chronic treatment that could induce insulin resistance (e.g., corticosteroids, rifampicin, isoniazid—used to treat tuberculosis, olanzapine, risperidone—used to treat psychotic conditions, or protease inhibitors—used to treat HIV infection).

### 2.3. Measurements

Maternal postpartum body weights were measured using electronic medical scales and RADWAG type WPT100/2000 to the nearest 100 g. To evaluate insulin resistance markers, peripheral venous blood for laboratory tests was taken from the mothers in the morning after overnight fasting using the BD Vacutainer blood-collecting system (Becton Dickinson, NJ, USA). The enzymatic method was used to determine fasting glucose (mg/dL) and an immonassay to measure insulin levels (μU/mL) and glycated hemoglobin (HbA1c; in %). Measurements were obtained in the hospital laboratory using Abbott analyzers (Abbott Laboratories, Chicago, IL, USA) and served to calculate the Homeostatic Model Assessment–Insulin Resistance (HOMA-IR) and the Quantitative Insulin Sensitivity Check Index (QUICKI). The HOMA-IR and QUICKI were calculated using the following formulas, respectively: (fasting insulin (μIU/I) × fasting glucose (mg/dL))/405 and 1/(log(fasting insulin μIU/I) + log (fasting glucose mg/dL)).

### 2.4. Data Collection

Data regarding the course of pregnancy, maternal antenatal history, and course of labor and puerperium were based on maternal medical records and personal questionnaires collected at the time of enrollment. Medical records were used to evaluate changes in maternal body weight during pregnancy. The categories of maternal gestational weight gain were set in accordance with guidelines published by the American College of Obstetrician and Gynecologist Committee Opinion [22] and described in this study as below, within, or above recommendations. A classification system was devised based on the maternal pre-pregnancy body mass index (BMI), delineating ranges for underweight, normal weight, overweight, and obese women.

### 2.5. Statistical Analysis

Statistical analysis was performed using STATISTICA 13.3 (StatSoft, Inc., Tulsa, OK, USA), Microsoft Excel for Microsoft 365 (Microsoft, Redmond, WA, USA), and R version 3.6.2 (R Core Team, 2013. R Foundation for Statistical Computing, Vienna, Austria). The data are presented in accordance with the following conventions: mean and standard deviation (SD), median and interquartile range (IQR), or number of cases and percentage, as appropriate. The results with *p* < 0.05 were considered significant. The demographic and clinical data from the study groups were subjected to analysis utilizing the one-way ANOVA, Kruskal–Wallis test, and Chi-square test, in accordance with the characteristics of the data and their distribution. The distributions of the studied variables were checked using the Shapiro–Wilk test. Univariate regression (generalized linear model) was used to assess the effect of selected maternal factors on postpartum markers of insulin resistance (HOMA-IR, QUICKI, and HbA1c). Furthermore, using the Marczewski–Steinhaus (M-S) taxonomic approach, cluster analysis was performed. The impact of the species was evaluated through the application of one-way ANOVA and the Kruskal–Wallis test. The taxonomic method was validated through the utilization of the expectation-maximization (E-M) algorithm [20,21]. Although alternative cluster analysis techniques are more widely known and more commonly used, the advantages of the M-S method led us to select it for our purposes. It focuses on the actual similarity of objects, allowing for more meaningful groupings with greater homogeneity of features within each cluster and greater heterogeneity of features between clusters. The method is more versatile because it does not require the data to be distributed in any particular way, nor does it require the assumption of linearity in the data, allowing flexible modeling of complex structures. Finally, the results are relatively easy to understand and interpret.

## 3. Results

### 3.1. General Characteristics of the Study Population

The general characteristics of the study group are shown in Table 1. The numbers of participants in the particular groups were as follows: *n* = 21 in the GDM G1 group, *n* = 29 in the GDM G2 group, and *n* = 20 in the non-GDM group. In the overall study group, the median duration of pregnancy at delivery was 39.0 (IQR 2.0) weeks (range 37–41 weeks), which was similar between the study groups (H (2, N = 70) = 3.246, *p* > 0.05). As described in detail in the previous manuscript, the study groups had similar results regarding neonatal gender, mode of delivery, parity, gravidity, as well as maternal age at delivery [22]. A review of the obstetric histories of all the mothers revealed that only *n* = 2 had delivered more than one or two babies in total. Both mothers were assigned to the GDM G2 group. The Chi-square test indicated that there was no significant difference in the distribution of fertility between the study groups (χ^2^ (2, N = 70) = 10.109, *p* = 0.257).

Also, neonatal anthropometric measurements and body composition were not statistically different between the study groups: weight at birth (F (2, 67) = 2.633, *p* > 0.05), length (F (2, 67) = 0.266, *p* > 0.05), and head circumference (F (2, 67) = 0.12, *p* > 0.05), as well as BMI (F (2, 67) = 1.859, *p* > 0.05) and PI (F (2, 67) = 2.792, *p* > 0.05) [14]. Regarding the body water compartments, no significant differences were found: TBW (F (2, 67) = 1.038, *p* > 0.05), TBW% (F (2, 67) = 1.440, *p* > 0.05), ECW (H (2, 70) = 2.903, *p* > 0.05), ICW (F (2, 67) = 1.053, *p* > 0.05), and E/I (H (2, N = 70) = 1.077, *p* > 0.05). Also, body fat: FBM (F (2, 67) = 2.758, *p* > 0.05), FBM% (F (2, 67) = 1.610, *p* > 0.05); and fat-free mass: LBM (F (2, 67) = 2.071, *p* > 0.05), LBM% (F (2, 67) = 2.174, *p* > 0.05) were comparable [22].

The obstetric history of the mothers in the study groups also did not differ with regard to the occurrence of GDM, neonatal hypertrophy, or macrosomia in the previous pregnancy (*p* > 0.05). However, newborns of mothers diagnosed with GDM were significantly more likely to be formula-fed than newborns of healthy mothers (χ^2^ (2, N = 70) = 8.784, *p* = 0.012). When other complications of pregnancy were taken into account, such as infections, abnormal fetal growth, excessive weight loss, nausea or vomiting, or a positive history of maternal hypothyroidism, hypertension, or nicotinism (before pregnancy), there was also no significant difference between the study groups (*p* > 0.05). However, GDM G1 and non-GDM mothers were found to have a significantly lower pre-pregnancy BMI and a lower incidence of obesity or overweight than GDM G2 mothers (H (2, N = 70) = 8.537, *p* = 0.014, and χ^2^ (4, N = 70) = 15.424, *p* = 0.004, respectively). Conversely, GDM G2 mothers had the lowest mean gestational weight gain—this parameter differed between the study groups in value and reference to recommendations (F (2, 67) = 12.923 *p* < 0.001, χ^2^ (4, N = 70) = 10.454, *p* = 0.033) (Table 1).

The median fasting glucose in the first trimester of pregnancy was comparable between the study groups (*p* > 0.05). There were however differences in the results of the OGTT: fasting glucose (H (2, 70) = 11.890, *p* = 0.003), 1 h post-OGTT glucose (H (2, 70) = 19.895, *p* < 0.001), 2 h post-OGTT glucose (H (2, 70) = 19.315, *p* = 0.001). When considering markers of insulin resistance in the first week postpartum, the study groups differed significantly in the results of HbA1c (H (2, N = 70) = 9.372, *p* = 0.009) and HOMA-IR (H (2, N = 70) = 6.177, *p* = 0.046), but the results of QUICKI were similar (*p* > 0.05) (Table 1).

### 3.2. Cluster Analysis

In the previous manuscript [21], the following factors were selected as best-fitting predictors of neonatal anthropometrics and body composition: study group, maternal history of hypothyroidism, and maternal weight gain during pregnancy. The taxonomic analysis resulted in the identification of four clusters of patients. The characteristics of the mothers in the clusters are presented in Table 2 below. In the current manuscript, the results of insulin resistance markers were compared between mothers assigned to the abovementioned clusters (Table 2).

To provide a complete description of the clustered patients, they were compared for data on selected maternal and neonatal characteristics, obstetric history, and maternal medical interview. The *n* = 2 mothers who had delivered more than two infants in total were assigned to “Cluster 3”. The distribution of fertility was similar between the clusters (χ^2^ (2, N = 70) = 3.117, *p* = 0.927). The results are presented in Table 3.

Concerning maternal gestational interview, clusters differed in incidence of excessive weight loss, which was the most frequent in “Cluster 2” and affected 37% of those mothers (χ^2^ (3, N = 70) = 10.533, *p* = 0.015). The next significant differences were in the results of glucose screening tests during pregnancy. Mothers in “Custer 1” were found to have the highest fasting glucose levels in both the first (H (2, 70) = 13.002, *p* = 0.005) and second/third trimesters of pregnancy (H (2, 70) = 10.389, *p* = 0.016). Glucose levels at 1 h post-OGTT were also significantly different between groups (H (2, 70) = 9.088, *p* = 0.028), but the higher median values were in “Cluster 2”. Glucose levels at 2 h post-OGTT were comparable between clusters (*p* > 0.05). For the clusters, the results of the HOMA-IR and the QUICKI did not show any differences in the first week after delivery (*p* > 0.05). Conversely, HbA1c results varied significantly (H (2, 70) = 19.935, *p* < 0.001). Multiple comparisons of IR markers between clusters are presented in Table 4.

## 4. Discussion

In the current study, the study groups correspond to the degree of glucose intolerance—the higher the glucose intolerance, the more intensive the glucose monitoring and the more specific the treatment. The target of GDM treatment is to keep blood glucose levels within normal limits to prevent adverse outcomes for both mother and baby. This can be achieved through diet, exercise, daily blood glucose monitoring, and insulin injections. If diet and exercise alone do not control blood glucose within 2 weeks, insulin should be given along with lifestyle changes and physical activity [2,23]. The findings of the present study are consistent with the above-mentioned assumptions—GDM G2, thus insulin-treated mothers, had the lowest gestational weight gain but the highest values of fasting glucose in the first and second/third trimesters, 1 h post-OGTT glucose, as well as HbA1c and HOMA-IR. When comparing study groups, these results were significantly different. Apart from the highest degree of glucose intolerance, GDM G2 mothers had similar pregnancy outcomes and incidence of selected complications (e.g., maternal hypertension, infections, adverse obstetric history, abnormal fetal growth, neonatal anthropometrics, neonatal congenital defects, and respiratory distress) to the mothers in the other two groups. This observation can result from adequate treatment and monitoring. The fact that only two of the mothers in the GDM G2 group had given birth to more than two children did not affect the results obtained. The number of these multiparous patients was insufficient to enable determination to be made regarding the relationship between high fertility (more than two childbirths in total) and the results obtained. All patients with GDM in the current study were offered dietary and lifestyle counseling and self-monitoring of blood glucose. The exception was the rate of exclusive breastfeeding, which was markedly lower. In general, women with GDM have less favorable breastfeeding results than women without GDM, despite similar rates of breastfeeding initiation. Evidence suggests that they are more likely to have delayed onset of lactogenesis II. Since women with GDM experience more difficulty in producing adequate milk volumes than those without diabetes, infants of mothers with GDM are more likely to be introduced to fluids other than human milk (e.g., formula) than infants of non-diabetic mothers [24,25,26].

As previously reported, only six mothers developed GDM in the previous pregnancy, three in GDM G1 and three in GDM G2. This observation had no impact on the results obtained between study groups and between clusters. Furthermore, the results of glucose levels in pregnancy and IR markers postpartum in these mothers were not distinctive. Considering the rates of cesarean sections, they were comparable between study groups and surprisingly high—over 70% of cases. However, in the authors’ hospital, the rate of cesarean sections is generally high, with indications set by obstetricians. The authors, as neonatologists, do not interfere with maternal management; thus, we report the above incidence resulting from lack of progress of labor, perinatal complications, and previous cesarean section performed on the mother—based on medical records. Given the lack of variation in the rate of cesarean sections between study groups and between clusters, the authors conclude that this did not impact the results obtained.

Gestational weight gain is one of the most important determinants of insulin resistance and adverse pregnancy outcomes [27]. Therefore, weight gain targets during pregnancy are stricter for participants with a higher pre-pregnancy BMI [2,22]. The use of the taxonomic method in the statistical analysis allowed a clear differentiation of the patients, not only on the basis of the degree of the disorders of glucose metabolism but also according to the gestational weight gain. As the identification of the best-fitting factors was primarily related to the neonatal body composition, maternal hypothyroidism was found to be the third significant factor [21]. To summarize, “Cluster 1” and “Cluster 2” concern GDM mothers, whereas “Cluster 3” and “Cluster 4” non-GDM mothers are differentiated by weight gain in pregnancy and maternal thyroid gland dysfunction.

Glycated hemoglobin provides information on the estimate of glucose concentration in the last 2–3 months of pregnancy [23,28]. Cluster analysis confirmed that GDM mothers had a higher percentage of HbA1c than non-GDM mothers: “Cluster 1” vs. “Cluster 4”; “Cluster 2” vs. “Cluster 3”; and “Cluster 2” vs. “Cluster 4”. However, mothers in “Cluster 1” (GDM mothers with normal thyroid gland function) had similar results as mothers in “Cluster 3” (non-GDM mothers with normal thyroid gland function). These two clusters differed in gestational weight gain among mothers, with the highest result in “Cluster 3”. Among non-GDM mothers (“Cluster 3” vs. “Cluster 4”) and among GDM mothers (“Cluster 1” vs. “Cluster 2”), HbA1c was similar. The post hoc analysis did not identify any statistically significant differences in HOMA-IR and QUICKI between the clusters. The above results may highlight the importance of gestational weight gain in the pathogenesis of insulin resistance. The only significant difference in post hoc analysis of results of glucose screening tests concerns fasting glucose level in the first trimester between “Cluster 1” and “Cluster 2”—mothers affected by hypothyroidism (“Cluster 2”) had significantly higher glucose level. It is known that thyroid hormones affect glucose homeostasis; therefore, hypothyroidism may alter metabolism rate, leading to obesity and insulin resistance [29,30]. In the current study, adequate management of GDM and/or hypothyroidism may have contributed to the lack of differences in OGTT results and IR markers in the first week postpartum between “Cluster 1” and “Cluster 2”, as well as between “Cluster 3” and “Cluster 4”. However, “Cluster 2” and “Cluster 3” differed in terms of 1 h post-OGTT glucose level (*p* < 0.05). In addition, mothers in these two clusters had the most divergent characteristics: “Cluster 2”—GDM with a positive history of hypothyroidism and average weight gain in pregnancy, whereas “Cluster 3”—non-GDM with a negative history of hypothyroidism and the highest gestational weight gain.

Taking into account the post hoc analysis of neonatal results, “Cluster 2” and “Cluster 3” differed significantly in neonatal birth weight and fat body mass: mean (SD) birth weight of 3.77 (0.41) kg and mean (SD) FBM of 0.34 (0.1) kg in “Cluster 3” vs. mean (SD) birth weight of 3.35 (0.51) kg and mean (SD) FBM of 0.25 (0.1) kg in “Cluster 2” [21]. Based on these results, it can be assumed that adequate glycemic control, thyroid hormone function, and gestational weight gain within recommended limits will ensure normal fetal growth. However, excessive gestational weight gain, even in healthy mothers, will significantly alter fetal development.

The assessment of neonatal body composition was conducted using a technique of bioimpedance analysis (BIA), which was selected due to its accessibility, portability, non-invasive nature, and expediency. A review of the literature suggests that BIA is an accurate and reproducible method for measuring body compartments in infants and young children [31,32]. However, the “gold standard” for the assessment of infant body composition remains the air displacement plethysmography system, which employs whole-body densitometric principles to ascertain body components (fat and fat-free mass) [31,32]. The method of BIA used in the current study therefore carries a risk of inaccuracy.

Surprisingly, non-GDM mothers exhibited the highest weight gain during gestation, with a high incidence of weight gain above recommendations for pre-gestational BMI. In our opinion, the results should also be verified between groups with gestational weight gain within normal ranges.

The findings that link insulin resistance with gestational weight gain (GWG) and hypothyroidism in patients with GDM have the potential to significantly influence postpartum screening and care by highlighting the risks of metabolic and thyroid dysfunction in the postpartum period [33,34]. In patients with GDM who have gained a greater amount of weight during pregnancy, more frequent assessments of thyroid function may be advantageous in the postpartum period. This is because these patients may be at an elevated risk of developing persistent thyroid dysfunction [35]. The early identification of postpartum hypothyroidism may prove beneficial in the prevention of further metabolic issues—hypothyroidism can contribute to ineffective glycemic control [36]. In addition, patients with excessive GWG might benefit from early, individualized postpartum weight management support. Given the bidirectional relationship between hypothyroidism and weight gain, it is plausible that postpartum weight management may prove an effective strategy for reducing the risk of thyroid dysfunction and improving metabolic health [37,38].

The presence of GWG and hypothyroidism in patients with GDM is associated with an increased risk of developing type-2 diabetes [33]. It is already standard practice to screen for glucose tolerance in patients with GDM in the postpartum period. However, findings on GWG and thyroid function may suggest that these assessments should be extended over a longer period [33,34,35,38].

The current findings may prompt healthcare organizations to develop guidelines that are specifically focused on postpartum thyroid screening and insulin resistance monitoring in women with GDM and high GWG. The establishment of clear protocols could facilitate the standardization of care and ensure that these women receive the requisite follow-up. Further research should concentrate on the development of effective, individualized screening and educational programs, encompassing body weight management and early detection of glucose metabolism and thyroid dysfunction disorders in the postpartum period.

The principal constraint of this study is the paucity of subjects. While the outcomes offer a comprehensive overview of the issues addressed, it is necessary to corroborate these findings with a larger sample size. In addition, it is not possible to guarantee the replicability of the results. There is also a risk that assumptions made on the basis of the data obtained are inaccurate. The next limitation is the lack of assessment of IR markers during pregnancy, so it was not possible to compare results at different stages of pregnancy and draw conclusions about changes in insulin resistance in the following weeks of pregnancy.

The patients who gave birth in the authors’ hospital came from different regions of the Lower Silesian Voivodeship. They were cared for by different obstetricians and received different advice and counseling. We cannot assess what influence the standardization of medical care in pregnancy would have on the results received by the patients and the pregnancy outcomes.

## 5. Conclusions

Maternal insulin resistance markers in the first week postpartum result from not only the degree of glucose metabolism disorders in pregnancy but also gestational weight gain. Other maternal conditions known to alter maternal metabolism, such as hypothyroidism, have a considerable influence on markers of insulin resistance. Gestational weight gain alone has a major and strong impact on pregnancy outcomes, particularly on fetal weight gain and development, and therefore the weight of the neonate at birth and the degree of adipose tissue accumulation. Clustering is a useful method for identifying risk factor groups.

The findings on gestational weight gain and hypothyroidism in gestational diabetes mellitus patients indicate a necessity for proactive postpartum management of thyroid and metabolic health. The implementation of targeted thyroid and glucose monitoring, personalized weight management, and enhanced patient education could enhance perinatal outcomes as well as long-term outcomes for these women.

## Figures and Tables

**Table 1:** Characteristics of the study groups.

	All Participants (*n* = 70)	GDM G1 (*n* = 21)	GDM G2 (*n* = 29)	non-GDM (*n* = 20)	*p* Value
Selected maternal characteristics					
Maternal age [years], Mean (SD)	32.7 (4.5)	33.9 (4.9)	32.1 (4.6)	32.5 (3.9)	0.363 ^b^
Maternal pre-pregnancy BMI, Median (IQR)	24.2 (6.4)	23.3 (3.5)	28.0 (6.8)	23.0 (2.8)	0.014 ^a^
Maternal classification by pre-pregnancy BMI, *n* (%)					
Normal	40 (57.1)	14 (66.7)	9 (31.0)	17 (85.0)	0.004 ^c^
Overweight	16 (22.9)	4 (19.0)	11 (38.0)	1 (5.0)	
Obese	14 (20.0)	3 (14.3)	9 (31.0)	2 (10.0)	
Maternal pregnancy-related weight gain [kg], Mean (SD)	11.5 (5.7)	10.2 (3.5)	9.2 (5.4)	16.2 (5.4)	<0.001 ^b^
Maternal pregnancy-related weight gain in reference to pre-pregnancy BMI, *n* (%)					
Below recommendations	18 (25.8)	7 (33.3)	9 (31.0)	2 (10.0)	0.033 ^c^
Within recommendations	26 (37.1)	10 (47.7)	11 (38.0)	5 (25.0)	
Above recommendations	26 (37.1)	4 (19.0)	9 (31.0)	13 (65.0)	
Fasting glucose in the first trimester of pregnancy [mg/dL], Median (IQR)	89.0 (14.2)	89.0 (17.0)	93.0 (9.2)	85.6 (18.9)	0.123 ^a^
Fasting glucose in the second/third trimester of pregnancy [mg/dL], Median (IQR)	92.0 (15.0)	90.0 (15.0)	95.0 (10.0)	85.3 (18.0)	0.003 ^a^
1 h post-OGTT glucose level [mg/dL], Median (IQR)	135.5 (82.0)	125.0 (81.0)	182.0 (46.0)	108.5 (32.7)	<0.001 ^a^
2 h post-OGTT glucose level [mg/dL], Median (IQR)	119.5 (50.0)	138.0 (53.0)	130.0 (47.0)	102.0 (12.0)	0.001 ^a^
Maternal HbA1c [%] in the first week postpartum, Median (IQR)	5.4 (0.4)	5.3 (0.4)	5.6 (0.3)	5.3 (0.5)	0.009 ^a^
Maternal HOMA-IR in the first week postpartum, Median (IQR)	0.66 (1.67)	0.55 (0.35)	0.80 (0.83)	0.49 (0.55)	0.046 ^a^
Maternal QUICKI in the first week postpartum, Median (IQR)	0.41 (0.13)	0.43 (0.05)	0.41 (0.06)	0.43 (0.07)	0.106 ^a^
Characteristics referring to a newborn infant					
Pregnancy duration [weeks], Median (IQR)	39.0 (2.0)	39.0 (2.0)	38.0 (1.0)	39.0 (2.0)	0.197 ^a^
Gender of newborn, *n* (%)					
Male	28 (40.0)	7 (33.3)	12 (41.4)	9 (45.0)	0.733 ^c^
Female	52 (60.0)	14 (66.7)	17 (58.6)	11 (55.0)	
Mode of delivery, *n* (%)					
Vaginal birth	20 (28.6)	7 (33.3)	7 (24.1)	6 (30.0)	0.766 ^c^
Cesarean section	50 (71.4)	14 (66.7)	22 (75.9)	14 (70.0)	
Neonatal weight at birth [kg], Mean (SD)	3.5 (0.5)	3.4 (0.6)	3.3 (0.4)	3.7 (0.4)	0.079 ^b^
Neonatal length at birth [cm], Mean (SD)	53.2 (2.8)	53.0 (3.5)	53.1 (2.5)	53.6 (2.3)	0.767 ^b^
Newborn’s head circumference [cm], Mean (SD)	34.7 (1.5)	34.5 (1.6)	34.8 (1.6)	34.7 (1.6)	0.887 ^b^
Percentile for neonatal birth weight, Median (IQR)	65.5 (44.0)	56.0 (45.0)	69.0 (49.0)	74.5 (35.5)	0.363 ^a^
Classification of neonatal birth weight, *n* (%)					
SGA	3 (4.3)	1 (4.7)	2 (6.9)	0 (0.0)	0.657 ^c^
AGA	56 (80.0)	17 (81.0)	23 (79.3)	16 (80.0)	
LGA	11 (15.7)	4 (14.3)	3 (13.8)	4 (20.0)	
Neonatal respiratory distress after birth, *n* (%)	6 (8.6)	1 (4.8)	4 (13.8)	1 (5.0)	0.422 ^c^
Congenital malformation in neonate, *n* (%)	4 (5.7)	4 (19.1)	0 (0.0)	0 (0.0)	0.006 ^c^
Method of feeding of a newborn, *n* (%)					
Exclusive breastfeeding	33 (47.1)	8 (38.1)	10 (34.5)	15 (75.0)	0.012 ^c^
Mixed feeding	37 (52.9)	13 (61.9)	19 (65.5)	5 (25.0)	
Maternal medical history during and before pregnancy					
Maternal hypertension, *n* (%)					
Chronic (onset before the pregnancy)	6 (8.6)	1 (4.8)	5 (17.2)	0 (0.0)	0.478 ^c^
Pregnancy induced	8 (11.4)	2 (9.5)	5 (17.2)	1 (5.0)	
None	56 (80.0)	18 (85.7)	19 (65.6)	19 (95.0)	
Maternal hypothyroidism, *n* (%)					
Chronic (onset before the pregnancy)	20 (28.6)	6 (28.6)	12 (41.4)	2 (10.0)	0.175 ^c^
Gestational (onset during the pregnancy)	13 (18.6)	5 (23.8)	4 (13.8)	4 (20.0)	
None	37 (52.9)	10 (47.6)	13 (44.8)	14 (70.0)	
Nicotinism before pregnancy, *n* (%)	22 (31.4)	6 (28.6)	11 (37.9)	5 (25.0)	0.597 ^c^
Nausea and vomiting, *n* (%)	14 (20.0)	5 (23.8)	6 (20.7)	3 (15.0)	0.774 ^c^
Initially excessive weight loss, *n* (%)	14 (20.0)	6 (28.6)	7 (24.0)	1 (5.0)	0.085 ^c^
VI, *n* (%)	6 (8.6)	1 (3.5)	4 (19.1)	1 (5.0)	0.121 ^c^
UTI, *n* (%)	6 (8.6)	3 (14.3)	3 (10.3)	0 (0.0)	0.239 ^c^
Obstetric history					
Number of pregnancies, Median (IQR)	2.0 (1.0)	2.0 (2.0)	2.0 (1.0)	2.0 (1.0)	0.520 ^a^
Number of deliveries, Median (IQR)	2.0 (1.0)	1.0 (1.0)	1.0 (1.0)	2.0 (1.0)	0.395 ^a^
Developed GDM in previous pregnancy, *n* (%)	6 (8.6)	3 (14.3)	3 (10.3)	0 (0.0)	0.238 ^c^
Delivered a neonate with body weight > 4 kg in previous pregnancy, *n* (%)	6 (8.6)	3 (14.3)	1 (3.5)	2 (10.0)	0.387 ^c^
Delivered a hypertrophic neonate in previous pregnancy, *n* (%)	7 (8.6)	2 (9.5)	2 (6.9)	3 (15.0)	0.657 ^c^
Delivered a hypotrophic neonate in previous pregnancy, *n* (%)	0 (0.0)	0 (0.0)	0 (0.0)	0 (0.0)	1.0 ^c^

^a^—Kruskal–Wallis test; ^b^—one-way ANOVA; ^c^—Chi-square test; SD—standard deviation; IQR—interquartile range.

**Table 2:** The following table presents the comparison of characteristics of mothers in clusters, based on the results of a one-way ANOVA test.

Cluster	*n*	Study Group			History of Hypothyroidism			Weight Gain During Pregnancy [kg] (Mean ± SD)
		GDM G1 (*n*)	GDM G2 (*n*)	Non-GDM (*n*)	Chronic (*n*)	Gestational (*n*)	None (*n*)	
1	23	10	13	0	0	0	23	11.1 ± 5.7
2	27	11	16	0	18	9	0	11.2 ± 5.7
3	14	0	0	14	0	0	14	16.5 ± 5.9
4	6	0	0	6	2	4	0	15.6 ± 1.7
F statistic	-	n/a			n/a			8.31
*p* value	-	n/a			n/a			<0.001

n/a—not applicable.

**Table 3:** Characteristics of clustered patients.

	Cluster 1 (*n* = 23)	Cluster 2 (*n* = 27)	Cluster 3 (*n* = 14)	Cluster 4 (*n* = 6)	*p* Value
Selected maternal characteristics					
Maternal age [years], Mean (SD)	32.9 (4.9)	32.7 (4.5)	33.8 (4.2)	29.7 (3.0)	0.301 ^b^
Maternal pre-pregnancy BMI, Median (IQR)	24.3 (5.8)	26.8 (7.8)	23.1 (2.7)	22.1 (3.5)	0.246 ^a^
Maternal classification by pre-pregnancy BMI, *n* (%)					
Normal	14 (60.9)	11 (40.7)	10 (71.4)	5 (83.3)	0.156 ^c^
Overweight	7 (30.4)	8 (29.6)	1 (7.1)	0 (0.0)	
Obese	2 (8.7)	8 (29.6)	3 (21.4)	1 (16.7)	
Maternal pregnancy-related weight gain [kg], Mean (SD)	11.1 (5.7)	11.2 (5.7)	16.5 (5.9)	15.6 (1.7)	<0.001 ^b^
Maternal pregnancy-related weight gain in reference to pre-pregnancy BMI, *n* (%)					
Below recommendations	3 (13.0)	10 (37.0)	4 (28.6)	1 (16.7)	0.417 ^c^
Within recommendations	8 (34.7)	10 (37.0)	6 (42.8)	2 (33.3)	
Above recommendations	12 (52.3)	7 (26.0)	4 (28.6)	3 (50.0)	
Fasting glucose in the first trimester of pregnancy [mg/dL], Median (IQR)	147.0 (43.0)	92.0 (11.0)	100.9 (13.1)	85.9 (3.2)	0.005 ^a^
Fasting glucose in the second/third trimester of pregnancy [mg/dL], Median (IQR)	119.0 (31.3)	94.0 (13.0)	95.0 (16.0)	83.8 (12.2)	0.016 ^a^
1 h post-OGTT glucose level [mg/dL], Median (IQR)	85.0 (10.2)	177.0 (70.0)	116.5 (16.0)	125.5 (45.7)	0.028 ^a^
2 h post-OGTT glucose level [mg/dL], Median (IQR)	89.0 (9.1)	136.0 (58.0)	106.5 (23.0)	115.3 (33.1)	0.219 ^a^
Maternal HbA1c [%] in the first week postpartum, Median (IQR)	5.5 (0.5)	5.6 (0.4)	5.2 (0.3)	5.0 (0.5)	<0.001 ^a^
Maternal HOMA-IR in the first week postpartum, Median (IQR)	0.66 (0.75)	0.65 (0.63)	0.75 (0.63)	0.39 (0.32)	0.274 ^a^
Maternal QUICKI in the first week postpartum, Median (IQR)	0.41 (0.07)	0.40 (0.06)	0.41 (0.08)	0.46 (0.05)	0.347 ^a^
Characteristics referring to a newborn infant					
Pregnancy duration [weeks], Median (IQR)	39.0 (2.0)	39.0 (2.0)	38.5 (1.0)	39.0 (2.0)	0.816 ^b^
Gender of newborn, *n* (%)					
Boys	12 (52.2)	9 (33.3)	6 (42.9)	1 (16.7)	0.344 ^c^
Girls	11 (47.8)	18 (66.7)	8 (57.1)	5 (83.3)	
Mode of delivery, *n* (%)					
Vaginal birth	7 (30.4)	6 (22.2)	5 (35.7)	2 (33.3)	0.804 ^c^
Cesarean section	16 (69.6)	21 (77.8)	9 (64.3)	4 (66.7)	
Neonatal weight at birth [kg], Mean (SD)	3.5 (0.6)	3.4 (0.5)	3.4 (0.4)	3.6 (0.4)	0.711 ^b^
Neonatal length at birth [cm], Mean (SD)	53.4 (3.3)	53.0 (2.6)	53.6 (2.2)	52.3 (2.6)	0.776 ^b^
Percentile for neonatal birth weight, Median (IQR)	69.0 (54.0)	56.0 (60.0)	63.0 (25.0)	68.0 (31.0)	0.798 ^a^
Classification of neonatal birth weight, *n* (%)					
SGA	1 (4.3)	1 (3.7)	1 (7.1)	0 (0.0)	0.961 ^c^
AGA	18 (78.3)	21 (77.8)	12 (85.8)	5 (83.3)	
LGA	4 (17.4)	5 (18.5)	1 (7.1)	1 (16.7)	
Neonatal respiratory distress after birth, *n* (%)	1 (8.7)	4 (14.8)	1 (7.1)	0 (0.0)	0.482 ^c^
Congenital malformation in neonate, *n* (%)	1 (8.7)	3 (11.1)	0 (0.0)	0 (0.0)	0.432 ^c^
Method of feeding of a newborn, *n* (%)					
Exclusive breastfeeding	11 (47.8)	8 (29.6)	9 (64.3)	5 (83.3)	0.433 ^c^
Mixed feeding	12 (52.2)	19 (70.4)	5 (35.7)	1 (16.7)	
Maternal medical history during and before pregnancy					
Maternal hypertension, *n* (%)					
Chronic (onset before the pregnancy)	2 (8.7)	1 (3.7)	0 (0.0)	0 (0.0)	0.594 ^c^
Pregnancy induced	2 (8.7)	5 (18.5)	1 (7.1)	0 (0.0)	
None	19 (82.6)	21 (77.8)	13 (92.9)	6 (100.0)	
Maternal hypothyroidism, *n* (%)					
Chronic (onset before the pregnancy)	0 (0.0)	18 (66.7)	0 (0.0)	2 (33.3)	<0.001 ^c^
Gestational (onset during the pregnancy)	0 (0.0)	9 (33.3)	0 (0.0)	4 (66.7)	
None	23 (100.0)	0 (0.0)	14 (100.0)	0 (0.0)	
Nicotinism before pregnancy, *n* (%)	9 (39.1)	10 (37.0)	3 (21.4)	0 (0.0)	0.219 ^c^
Nausea and vomiting, *n* (%)	3 (13.0)	7 (25.9)	3 (21.4)	1 (16.7)	0.719 ^c^
Initially excessive weight loss, *n* (%)	1 (4.3)	10 (37.0)	1 (7.1)	2 (33.3)	0.015 ^c^
VI, *n* (%)	2 (8.7)	0 (0.0)	3 (21.4)	1 (16.7)	0.112 ^c^
UTI, *n* (%)	1 (4.3)	5 (18.5)	0 (0.0)	0 (0.0)	0.121 ^c^
Obstetric history					
Number of pregnancies, Median (IQR)	2.0 (2.0)	2.0 (1.0)	2.0 (2.0)	1.0 (1.0)	0.158 ^a^
Number of deliveries, Median (IQR)	2.0 (1.0)	1.0 (1.0)	2.0 (1.0)	1.0 (1.0)	0.344 ^a^
Developed GDM in previous pregnancy, *n* (%)	2 (8.7)	3 (11.1)	1 (7.1)	0 (0.0)	0.844 ^c^
Delivered a neonate with body weight > 4 kg in previous pregnancy, *n* (%)	0 (0.0)	4 (14.8)	1 (7.1)	1 (16.7)	0.257 ^c^
Delivered a hypertrophic neonate in previous pregnancy, *n* (%)	2 (8.7)	3 (11.1)	2 (14.3)	0 (0.0)	0.793 ^c^
Delivered a hypotrophic neonate in previous pregnancy, *n* (%)	0 (0.0)	0 (0.0)	0 (0.0)	0 (0.0)	1.0 ^c^

^a^—Kruskal–Wallis test; ^b^—one-way ANOVA; ^c^—Chi-square test; SD—standard deviation; IQR—interquartile range.

**Table 4:** Comparative analysis between clusters (post hoc Dunn’s test *p* values) for selected parameters of maternal markers of insulin resistance and maternal glucose levels in pregnancy.

Difference Between Clusters	Maternal HbA1c [%]	Maternal HOMA-IR	Maternal QUICKI	Fasting Glucose in the First Trimester of Pregnancy [mg/dL]	Fasting Glucose in the Second/Third Trimester of Pregnancy [mg/dL]	1 h Post-OGTT Glucose Level [mg/dL]	2 h Post-OGTT Glucose Level [mg/dL]
1–2	0.944	1.0	1.0	0.013	0.036	0.565	0.410
1–3	0.534	1.0	1.0	0.289	0.391	0.748	1.0
1–4	0.016	0.447	0.501	1.0	1.0	1.0	1.0
2–3	0.018	1.0	1.0	1.0	1.0	0.023	1.0
2–4	<0.001	0.520	0.527	0.362	0.061	0.156	0.816
3–4	0.607	0.819	1.0	0.935	0.572	1.0	1.0

## Data Availability

The original contributions presented in the study are included in the article, further inquiries can be directed to the corresponding author.
